# Process Evaluation and Investigation of Cultural Adaptations for an Online Parent-Based Intervention Using a Mixed-Method Approach

**DOI:** 10.1007/s10935-024-00781-3

**Published:** 2024-04-28

**Authors:** Reed M. Morgan, Constanza Trejo, Bradley M. Trager, Sarah C. Boyle, Ina M. Koning, Joseph W. LaBrie

**Affiliations:** 1 Department of Psychology, Fordham University, New York, USA; 2 Department of Psychological Science, Loyola Marymount University, 1 LMU Drive Suite 4700, Los Angeles, CA 90045 USA; 3 Psychology Department of Health and Student Welfare, Pontifical Catholic University of Chile, Santiago, Chile; 4 Department of Clinical Child and Family Studies, VU University Amsterdam, Amsterdam, Netherlands

**Keywords:** Process evaluation, Parent-based intervention, Adolescence, mHealth, Alcohol, Cross-cultural

## Abstract

Most alcohol intervention research focuses on program efficacy, yet few studies have investigated the acceptability of a program’s design and implementation to the target population or adapting existing alcohol interventions to different populations. To address these gaps in the literature, we (1) examined participant responsiveness to and implementation quality of FITSTART+, a web-app delivered parent-based alcohol intervention designed for incoming first-year college students in the United States, and (2) gathered feedback on how this intervention could be adapted to other populations of parents. A sample of U.S. parents of 17–20-year-old first-year college students (*N* = 109) participated in FITSTART+ during their child’s first year of college and completed a survey about parents’ responsiveness to the app and its quality. Next, a sample of non-U.S. parents of adolescents aged 13 to 19 (*N* = 44) participated in one of 11 focus groups in which they briefly explored the app and then discussed how it could be adapted to be applicable and culturally relevant for them and their context. Results revealed that U.S. parents rated the intervention’s quality as high and parents were responsive to the web-app’s content, but some did not visit one of the most critical aspects of the intervention (i.e., alcohol-related parenting resources). Non-U.S. participants provided a range of suggestions for adapting the intervention to their context, which varied by culture. Results identify areas for improvement, particularly regarding the use of alcohol-related parenting resources, in this intervention and for web-delivered PBIs more broadly.

Alcohol use among adolescents is widespread (Johnston et al., 2019; World Health Organization [WHO], 2018). Early initiation of drinking poses a serious public health issue, with significant behavioral, clinical, social, and economic consequences (Substance Abuse and Mental Health Services Administration [SAMHSA], 2021; U.S. Department of Health and Human Services, 2016; WHO, 2018). Thus, the implementation and evaluation of effective evidence-based interventions aimed at preventing or delaying alcohol consumption among adolescents is an urgent matter. Parent-based interventions (PBIs) are effective in preventing high-risk alcohol consumption by improving parent-child communication, rule setting, and monitoring (e.g., Koning et al., 2009, 2011; LaBrie et al., 2016, 2022; Turrisi et al., 2001, 2009). Although there is clear evidence that PBIs can help to curb high-risk drinking, studies exploring the design and implementation of these interventions (i.e., process evaluations) are scarce—particularly for progressive web-based interventions—which complicates efforts to understand their outcomes (Foxcroft & Tsertsvadze, 2012). It is crucial to evaluate how well a web-based PBI is implemented among its target population. When considering disseminating an effective intervention to populations other than the one it was originally designed for, it is also crucial to evaluate how it can be appropriately modified to appeal to other social contexts. The current study examined parents’ perceptions of responsiveness to and quality of a web-based college drinking PBI for United States parents of incoming college students to inform modifications that could be made to improve its effectiveness with the target population. We also conducted focus groups with parents of adolescents from outside the U.S. to explore possible adaptations that could make the intervention relevant to those contexts.

## Process Evaluations for Digital Interventions

The study of implementation, also referred to as process evaluation, has been defined as “how well a proposed program or intervention is put into practice” (Durlak, 1998, p. 5). Durlak and DuPre (2008) argue that there are at least eight dimensions of implementation: fidelity, dosage, quality, responsiveness, program uniqueness, monitoring of comparison conditions, reach, and modification. Process evaluations tend to focus on a single dimension, creating a gap in our understanding of how different aspects of implementation interact with each other (Berkel et al., 2011; Durlak & DuPre, 2008; Humphrey et al., 2016; Tolan et al., 2014). In evaluating web-based interventions, certain dimensions are irrelevant (i.e., fidelity and dosage) while others are more crucial to the intervention’s success (i.e., quality and responsiveness). Studying responsiveness to intervention content, which includes participants’ level of engagement with intervention materials, is important because lower responsiveness can translate into weaker effects (i.e., if participants do not engage with the content, the intervention is unlikely to be effective). Responsiveness is of particular importance for—and must be high in—web-based interventions because, in the absence of a facilitator ensuring that all intervention content is delivered and participants remain engaged, participants are responsible for their own engagement with the material. To ensure adequate responsiveness, the intervention’s content must be of high enough quality to captivate and maintain participants’ interest and attention. Lower quality interventions (e.g., those with content that is difficult to find or presented in an unattractive format) should elicit lower responsiveness. Web-based interventions perceived by participants as being higher quality (e.g., easy to use; content is relevant, easily accessible, and presented in an attractive format) should have better responsiveness, which, in turn, should increase the effectiveness of the intervention (Berkel et al., 2011). Indeed, quality has been shown to affect how responsive individuals are to intervention content (e.g., LoCasale-Crouch et al., 2016; Rohrbach et al., 2010; Vandelanotte et al., 2021).

Examination of the literature would reveal studies on user experience and satisfaction with alcohol-related web-based interventions are scarce. One study that examined a smartphone intervention for alcohol use disorder emphasized the importance of increasing user engagement as a key issue for technology-based interventions (Giroux et al., 2014). This is supported by a recent review on internet-based eating disorder interventions among adults (Zeiler et al., 2021). However, to our knowledge, there are no studies that examine parents’ perceptions of and experiences with web-based PBIs. Understanding parents’ responsiveness to and perceived quality of a PBI will help to determine ways to further improve the effectiveness of these programs.

## **Adaptation of an** Intervention **to New Populations**

The successful transfer of an intervention from one context/culture to another rests partially on whether the program is adapted to meet the needs of the new target population. According to Durlak and Dupre (2008) and Stirman et al. (2013), to ensure the successful transferability of an intervention, there should be content modifications to improve the cultural fit and respond to the local value systems, as well as contextual modifications that do not alter the core elements of the intervention like the delivery strategy. Guidance about how to conduct such a process includes a systematic review of interventions re-evaluated in new contexts, qualitative interviews with stakeholders, and an effort to identify areas of consensus and uncertainties that might determine if the intervention is contextually contingent (Brownson et al., 2022; Evans et al., 2019). Implementing an existing PBI in a different country has been carried out successfully for interventions like the Strengthening Families Program (e.g. Segrott et al., 2022; Skärstrand et al., 2014) and Incredible Years (Hutchings et al., 2012; Larsson et al., 2009). However, these programs are examples of interventions that were merely translated into another language and did not involve the targeted parents in its development, nor did they undergo an adaptation and modification process, which may have hampered their overall effectiveness. Before deciding to transfer and implement an existing intervention model, it is necessary to critically evaluate the intervention’s components, strengths, and shortcomings (Brownson et al., 2022; Movsisyan et al., 2019; Park et al., 2022). This is especially true when considering the transferability of PBIs developed in the U.S., where the legal drinking age is much older than in other countries. Other cultural/contextual factors may also impact responsiveness to a digital intervention in a given country. For instance, availability of alcohol, predominant religious beliefs, societal norms and attitudes towards drinking, and acceptability of government and/or private campaigns targeting alcohol use may all affect implementation. Given this, the PBI content may not be entirely applicable to parents outside of the U.S., but the overall strategy (i.e., providing parenting-related resources via a web-app) may still be. To increase effectiveness of interventions, it is important to gain insight into how interventions should be culturally adapted (Ozge et al., 2024).

### FITSTART+ Intervention

The current study conducted a process evaluation and investigation of cultural adaptations of FITSTART+. FITSTART+ is a free, online, web-app-based college drinking PBI designed for U.S. parents of incoming college students (LaBrie et al., 2022, 2024). This intervention is innovative in two ways: (1) it is delivered online via a web-app, and (2) it incorporates personalized normative feedback (PNF) in the form of a parenting and alcohol quiz that is designed to motivate parents to engage with intervention content and implement risk-reducing behaviors such as disapproval toward underage drinking. It was developed using a person-centered approach (i.e., co-design) where the target population was directly involved in the intervention’s design and content selection via focus groups and surveys. This approach is considered a best practice when developing intervention content to ensure it is relevant and appropriate to the target population (LaMonica et al., 2022; Noorbergen et al., 2021; Perski & Short, 2021; Yardley et al., 2015). FITSTART+ has been given to two cohorts of parents and shown to be effective in the prevention of problematic alcohol consumption among first-year college students in the U.S. (LaBrie et al., 2022, 2024).

FITSTART+ is accessible via any device with an Internet connection (i.e., smartphone, tablet, or laptop/desktop computer). The website layout adapts to the device used, meaning that content is displayed in a way that properly fits the screen it is being viewed on. Parents can access FITSTART+ by creating a profile and logging in (i.e., provide their name, student’s sex, and number of children in/graduated from college on the FITSTART+ webpage). Once logged in, they are taken to the homepage. The two primary components of the intervention are the PNF quiz and the resource library. The PNF quiz consists of six questions. Three questions assess parents’ perceptions of how many drinks the typical student consumes per week during their first year in college, the maximum number of drinks the typical first-year parent would find acceptable for their student to consume on a single occasion during their first year in college, and the percent of first-year parents that talked to their student about drinking in the past month. Three parallel questions assess parents’ perceptions of their own student’s drinking, their own approval toward drinking, and their own communication with their student. Following the questions, results are presented using graphics which are accompanied by an audio explanation of the results. Consistent with social norms theory (Berkowitz, 2004), the PNF is designed to correct parents’ misperceptions, which, in turn, should motivate them to engage with intervention materials in the resource library, and motivate them to sustain and/or increase risk-reducing communication with their student (e.g., communicate disapproval toward underage drinking; Mallett et al., 2019; Napper, 2019). Illustrations of the FITSTART+ PBI can be found in LaBrie et al. (2024).

Following the PNF, parents are automatically redirected to a video that provides them with an explanation of the quiz and an overview of key concepts that they will encounter in the resource library. A list of recommended alcohol-related articles and a link to the resource library are provided at the end of the video. The resource library, which parents can also access via the homepage, includes several sections dedicated to college student drinking (College Drinking 101, Confronting Alcohol Myths), parenting (Parenting Around Alcohol, Having the Right Conversation, From Our Experts), and general information that was not central to PBI (e.g., Navigating the First Year). Material found in the alcohol- and parenting-specific sections was derived from Turrisi and colleagues’ (2001) Parent Handbook. To appeal to different types of parents, content was delivered in three formats: via writing, graphics, and videos.

## Current Study

The current study evaluated the implementation of FITSTART+ using two distinct parent samples. This dual sample study was designed to (1) multidimensionally examine implementation of the FITSTART+ intervention in the parent population for which it was specifically developed, and (2) gather feedback on how the intervention might be adapted for successful implementation in other parent populations. In the U.S. sample, the FITSTART+ intervention was fully implemented among parents of first-year college students as intended and parents completed a post-intervention feedback survey about their experiences using the app. In the non-U.S. sample, parents of adolescents residing in the Netherlands, Canada, Chile, and Germany, notably diverse in their cultural values and attitudes toward alcohol consumption, explored the U.S. version of the FITSTART+ web-app within focus groups to ascertain feasibility and acceptability, and to inform future cultural adaptations. The non-U.S. sample consisted of parents of adolescents younger than those in the U.S. sample because of the younger legal drinking ages in the surveyed countries relative to the U.S. Although FITSTART+’s content may not be entirely relevant to parents outside of the U.S. whose children are legally permitted to drink during the transition into college, delivering intervention content via a web-app and providing PNF are improvements over commonly disseminated psychoeducation-only PBIs and intervention content can be easily adapted for use with parents of adolescents from other countries.

## Method

### Procedure and Participants

All study procedures were approved by an Institutional Review Board.

#### U.S. FITSTART+ Implementation Sample

Parents of incoming non-international first-year students aged 17–20 at a private, mid-sized west coast university in the United States were invited via email to sign up for FITSTART+ (December 2020). The invitation described the program as a resource guide designed to help parents promote their students’ health and well-being during their first year of college. Parents who created a profile in the FITSTART+ intervention (*N* = 209; 81.8% female) were invited to complete an online feedback survey three months after the initial invitation to join the web-app. To increase ecological validity of parents’ use of FITSTART+, informed consent to use the app was embedded within the program’s terms and conditions, which all parents agreed to prior to creating an account. Parents provided digital informed consent to participate in the feedback survey via Qualtrics prior to beginning the survey. The survey took approximately 15 min to complete, and recruitment consisted of six invitation emails sent to parents over a six-week period. Parents who completed the survey were entered into a raffle for a $100 Amazon gift card. A total of 109 parents responded to the survey; those who participated did not differ from those who did not in terms of their sex, their student’s sex, or number of children in/graduated from college (*p*s > .05). See Table 1 for demographic information for the U.S. sample.

**Table 1:** Sample characteristics of the U.S. and non-U.S. samples

	U.S. sample		Non-U.S. sample	
	*M*/*n*	*SD*/%	*M*/*n*	*SD*/%
Gender				
Male	19	17.8%	7	12.9%
Female	88	82.2%	37	87.1%
Marital status				
Single	3	2.9%	0	0.0%
Married	93	89.4%	30	68.2%
Divorced	8	7.7%	13	29.5%
In a relationship but not married	-	-	1	2.3%
Age			48.9	5.13
40–49	18	17.3%	-	-
50–59	77	74.0%	-	-
60–69	8	7.7%	-	-
70+	1	1.0%	-	-
Race				
African American/Black	6	5.8%	-	-
Asian	11	10.6%	-	-
Caucasian/White	78	75.0%	-	-
Native Hawaiian/Pacific Islander	1	1.0%	-	-
Multiracial	3	2.9%	-	-
Other	5	4.8%	-	-
Ethnicity				
Hispanic/Latinx	13	12.5%	-	-
Not Hispanic/Latinx	91	87.5%	-	-

*Note*: U.S. sample *n*s ranged from 104–107 due to parents skipping demographic information questions. Dashes indicate questions that were not asked to that sample

#### Non-U.S. FITSTART+ Feedback Sample

Non-U.S. parents of adolescents aged 13–19 who could read and understand English were invited to participate in focus groups to identify ways FITSTART+ could be adapted to appeal to populations of parents outside of the U.S. (January–February 2021). Parents were recruited through the social network of research assistants studying at Utrecht University, and therefore the selection of the countries involved in this study was based on the nationality of the research team (i.e., convenience sampling method). After providing digital informed consent via Qualtrics, parents completed a brief online demographics survey and were asked to attend an online focus group with other parents from the same country in which they explored the FITSTART+ web-app for a minimum of 15 min and then discussed their opinions of the intervention. The online focus groups were conducted in each group’s native language by previously trained research assistants. The audio from all meetings was recorded and later transcribed and translated into English. A total of 44 parents attended one of 11 focus groups. There were five focus groups with Dutch parents (*n* = 18), three with Canadian parents (*n* = 13), two with Chilean parents (*n* = 8), and one with German parents (*n* = 5). See Table 1 for non-U.S. sample demographic information. Although parents from these countries were selected as a convenience sample, they nonetheless represent countries with elevated high-risk drinking among adolescents (WHO, 2018).^1^ The non-U.S. sample therefore represents countries that would stand to benefit greatly from web-based alcohol interventions.

### Measures

#### U.S. FITSTART+ Implementation Sample

In the U.S. sample, four key aspects assessing two main concepts of parents’ experience with FITSTART+ were measured in an online questionnaire: (1) Responsiveness (exploration and ease of use) and (2) Quality (quality of content and suggestions for improvement).

**Responsiveness.** Responsiveness was measured by two concepts: *exploration* and *ease of use*. To get an indication of parents’ *exploration* through the app, participants were asked how many times they visited FITSTART+, including the time they signed up (*1* to *7+*). Parents were then asked to indicate which of the following they did in the web-app: (a) *Viewed or read articles*, (b) *Watched videos*, (c) *Took the parenting and alcohol quiz*, (d) *Viewed other parents’ profiles*, and (e) *Viewed [their] own profile*. Parents then selected which resource library sections and post-PNF quiz recommended articles they recalled viewing; a dichotomous variable was created based on whether they did (1) or did not (0) report viewing any of the alcohol and/or parenting sections or post-PNF quiz recommended alcohol-related articles. Lastly, the percent of parents who completed the PNF quiz was computed using objective quiz data which were tied to each user’s FITSTART+ account.

Parents indicated the *ease of use* by rating how easy or difficult it was to sign up and create a profile in FITSTART+ from *Extremely easy* (1) to *Extremely difficult* (5) and whether or not they experienced any technical difficulties that affected their ability to access or use features in FITSTART+. Parents were also asked to rank FITSTART+’s usability/user-friendliness on a scale from 0 to 5.

**Quality.** Quality of implementation consisted of two concepts: *quality of content* and *suggestions for improvement*. To measure the *quality of content*, parents were asked to rank FITSTART+’s content, appearance/aesthetics, and overall experience on a scale from 0 to 5. Participants were also asked whether they would recommend FITSTART+ to parents of incoming first-year students. Then, they were asked to indicate whether they mentioned FITSTART+ to any of the following people: (a) *[Their] spouse/partner*, (b) *A parent of a college student (not [their] spouse/partner)*, (c) *A parent of a non-college student (not [their] spouse/partner)*, (d) *[Their] student*, (e) *Other*, or (f) *No one*. Parents were then asked if they planned on using FITSTART+ in the coming months. Lastly, parents were asked to respond to the open-ended question, *Please describe what you found most helpful about FITSTART+*.

*Suggestions for improvement* was measured by asking parents to indicate (a) whether they would be interested in a moderated forum/message board in the web-app that would allow parents to communicate with each other, (b) whether parents would have liked to receive email updates about new and recommended resources in FITSTART+, and (c) how often they would like to see new content. The first two questions were rated dichotomously, with answer options for the third ranging from *Daily* (1) to *Every couple of months* (6). Participants were also asked if they would prefer the resource information in the intervention to be presented as articles to read, videos to view, if they were indifferent, or if it depended on the content. Parents were also asked two open-ended questions to further probe suggestions for improvement: *Please provide any suggestions you may have that could help us improve the content, appearance, and usability of FITSTART+*; and *Please share any additional comments you might have about FITSTART+.*

#### Non-U.S. FITSTART+ Feedback Sample

Parents in the non-U.S. sample were asked about adaptations that could be made to the app to make it relevant to their cultural context. These focus group discussions allowed a deeper and more detailed understanding of the participants’ experiences and sometimes led to a conversation about specific aspects of the intervention’s design. Participants were asked a number of open-ended questions to facilitate conversation and receive feedback centered around three goals: to determine (1) if parents in different countries would use FITSTART+ or a similar PBI, (2) parents’ perceived benefits and barriers to using the intervention, and (3) what changes could be made to the app to make it more relevant to parents’ cultural contexts.

### Analytic Plan

Quantitative survey items were analyzed descriptively using SPSS version 27. Responses to open-ended survey questions were reviewed and coded for common themes. All focus group transcriptions were translated to English for analysis, and transcripts were then coded and analyzed using NVivo 12 pro software for qualitative data. Following the guidelines of Braun and Clarke (2022), thematic analysis was used to identify, analyze, and report patterns within the data. We used a deductive orientation of reflexive thematic analysis—the study’s research questions were used as a starting point to identify the commonly recurring themes across the data (Braun & Clarke, 2022). During the first round of coding, the participants’ country of origin was not considered to create a general overview of the data. Later, during the second round of coding, differences between countries were examined.

## Results

### U.S. FITSTART+ Implementation Sample

In-text results highlight key quantitative findings and all qualitative data; a full list of quantitative results can be found in Table 2 (responsiveness) and Table 3 (quality).

**Table 2:** U.S. sample responsiveness quantitative data

Exploration					
	**Range**	**M**	**SD**	**N**	**%**
*Including the time you signed up, approximately how many times did you visit FITSTART+?*	1–6	1.77	0.92		
*Please indicate what you did in FITSTART+*:					
* Viewed or read articles*				61	56.0%
* Watched videos*				28	25.7%
* Took the parenting and alcohol quiz*				59	54.1%
* Viewed other parents’ profiles*				15	13.9%
* Viewed your own profile*				30	27.8%
Parent reported visiting alcohol and/or parenting sections					
Yes				63	57.8%
No				46	42.2%
Objective PNF quiz completion data					
Did not complete quiz				30	27.5%
At least once				79	72.5%
2 + times				5	4.6%
**Ease of Use**					
	**Range**	**M**	**SD**	**N**	**%**
*How easy or difficult was it to sign up and create a profile in FITSTART+?*	1–5	1.58	0.81		
*Did you experience any technical difficulties that affected your ability to access or use features in FITSTART+?*					
* Yes*				10	9.6%
* No*				94	90.4%
*Please rank FITSTART + on the following*:					
* Usability/user friendliness*	0–5	4.02	0.97		

**Table 3:** U.S. sample quality quantitative data

Quality of Content					
	**Range**	**M**	**SD**	**N**	**%**
*Please rank FITSTART + on the following*:					
* Content*	0–5	3.64	1.05		
* Appearance/aesthetics*	0–5	3.98	0.95		
* Overall experience*	0–5	3.64	1.11		
*Would you recommend FITSTART + to parents of incoming first-year students?*					
* Yes*				83	88.3%
* No*				11	11.7%
*Did you mention FITSTART + to any of the following people?*					
* Your spouse/partner*				44	40.4%
* A parent of a college student (not your spouse or partner)*				5	4.6%
* A parent of a non-college student (not your spouse/partner)*				2	1.9%
* Your student*				34	31.2%
* No one*				40	37.0%
*Do you plan on using FITSTART + in the coming months?*					
* Yes*				61	63.5%
* No*				4	4.2%
* Not sure*				31	32.3%
**Suggestions for Improvement**					
	**Range**	**M**	**SD**	**N**	**%**
*Would you be interested in a moderated forum/message board in FITSTART + that would allow parents to communicate with each other?*					
* Yes*				65	68.4%
* No*				30	31.6%
*Would you have liked to receive email updates about new and recommended resources in FITSTART+?*					
* Yes*				84	88.4%
* No*				11	11.6%
*How often would you like to see new content on FITSTART+?*					
* A few times a week*				3	3.2%
* Once a week*				35	36.8%
* A couple of times a month*				29	30.5%
* Once a month*				25	26.3%
* Every couple of months*				3	3.2%
*Would you prefer the resource information in FITSTART + to be presented as…*					
* Articles that you read*				30	31.6%
* Videos that you watch*				9	9.5%
* I am indifferent*				43	45.3%
* It depends on the content*				13	13.7%

#### Responsiveness

**Exploration.** Including the time they signed up, participants reported signing in to FITSTART+ 1.77 times on average. Over half of parents self-reported viewing or reading articles (56.0%) and taking the parenting and alcohol quiz (54.1%). Interestingly, 57.8% reported visiting the alcohol/parenting-related resource library sections or viewing the post-PNF recommended alcohol articles—a slightly greater percentage than those who reported viewing any articles. Objective data show that the majority of parents took the quiz at least once (72.5%), with 4.6% taking it twice or more.

**Ease of Use.** Parents reported that signing up and creating a profile was easy, and the majority (90.4%) did not report any technical difficulties. The intervention’s usability/user-friendliness was also rated positively.

#### Quality

**Quality of Content.** The intervention’s content, appearance/aesthetics, and overall experience were all ranked well, and most of the sample (88.3%) indicated that they would recommend FITSTART+ to parents of incoming first-year students. The most frequently endorsed person participants mentioned FITSTART+ to was their spouse/partner (40.4%). Most parents (63.5%) indicated that they planned to use FITSTART+ in the coming months.

Forty-five parents (41.3%) responded to the open-ended question, *Please describe what you found most helpful about FITSTART+*. The most common answer theme was related to the articles and videos (*n* = 15): for example, *“Good information presented in an aesthetically pleasing way. Not overwhelming chunks of writing”*; *“The informational part of it, and how the information was presented.”* The second most common category was related to alcohol content (*n* = 14): *“[the] discussions and helpful tips around subject matters like drinking that may be different from when we were in college 30 years ago.”* Others commented about FITSTART+’s ease of use (*n* = 3): *“Easy to follow and understand”* and that it brought a sense of belonging (*n* = 3): *“Other parents to connect with.”* The remaining responses did not fit any common theme (e.g., *“I think the concept is a nice way to support parents particularly with first year college children”*).

**Suggestions for Improvement.** The majority of parents were in favor of a moderated forum/message board in FITSTART+ and receiving email updates about new and recommended resources. The most frequently endorsed option for how often parents would like to see new content on FITSTART+ was once a week (30.5%). More parents preferred the resource information to be presented as articles that you read (31.6%) than videos that you watch (9.5%); 45.3% indicated that they were indifferent and 13.7% indicated that it depended on the content.

For the open-ended question, *Please provide any suggestions you may have that could help us improve the content, appearance, and usability of FITSTART+*, 32 parents (29.4%) provided a valid response. The most commonly endorsed suggestion was to add more resources (*n* = 10): *“In addition to existing content, tips from parents with students already at [university] and have been through experiences such as moving their student on campus.”* The second most frequent suggestion for improvement was about organization (*n* = 8): *“The homepage doesn’t have enough content on it to orient me to all of the other information. Needs some sort of introduction about what the app includes”*; *“Help the users understand the purpose and intent of the site”*; *“Be more specific about what to expect from FITSTART+ and how to use it.”* Other categories included technical difficulties (*n* = 4), increasing communication (*n* = 3): *“Possibly use reminder emails to announce updates, etc.”*; the ability to connect with other parents (*n* = 3): *“It will be great to be able to connect with parents of students in the same academic programs”*; and other (*n* = 4).

The final open-ended question, which asked for any additional comments about FITSTART+, received valid responses from 22 parents (20.2%). The most frequent theme was positive feedback (*n* = 8): *“FITSTART+ is great for parents with students leaving for college. Wish I had found it sooner.”* Other themes included organization recommendations (*n* = 6): *“Looks like a great site with lots of info, it just needs to be better organized and then it will be a valuable tool for me”*; resource recommendations (*n* = 6): *“I would like to see FITSTART+ possibly expanded to parents of all years… I have a neighbor whose son is a [high school] senior and it was good to know about what to expect about off campus living and [university] forms, etc.”*; and communication recommendations (*n* = 2): *“It would be helpful to advertise the purpose and intent of this utility.”*

### Non-U.S. FITSTART+ Feedback Sample

Results are organized based around the three goals of the focus groups.

#### Parents’ Willingness to Use FITSTART+

Parents were overall apprehensive about using FITSTART+ themselves but most agreed that the intervention could be helpful to other parents in their country, especially for those with smaller support networks. Many parents who explicitly mentioned that they would not use the web-app said it did not apply to their situation, either because their children did not drink or they lived at home: *“I don’t think I would use the app in all honesty, because at this moment it doesn’t affect me. Could I possibly use it in the future? I won’t say I won’t, because it may be an app that will comfort me at that time” (Parent 13, Canada).*

Canadian parents had the most positive opinions of FITSTART+ and most frequently mentioned they would use it. Chilean parents had a more moderate opinion as they thought the web-app could be beneficial, but only if accompanied by a broader intervention about other health behaviors: *“I find potential in the app, but I think if the app by itself is not accompanied by a greater conversation about community issues, I don’t see much use for it.” (Parent 6, Chile).* German and Dutch parents were most critical of the web-app, and only three out of 23 participants from these countries said they would use the web-app if needed. German parents mentioned that they would be more inclined to use trusted and familiar sources for advice: *“If you were having sensitive issues with your teenager or young adult [such as] alcohol issues, you’d be much more inclined to touch base with someone who is very close to you personally and who knows your child also, so that you could [receive] very trusted advice on how to handle this”* (Parent 1, Germany). Parents from both countries also cited established organizations and the government as their preferred sources of information: *“in the Netherlands we have good foundations and good websites from the government where you can find reliable information”* (Parent 2, Netherlands); *“I don’t think that would be something we would ever pick up. I think we would be talking to our child and would be looking at different organizations if we felt there was an issue”* (Parent 5, Germany). Compared to parents from other countries, Dutch parents most frequently said that such an intervention was not necessary because they already openly discuss topics like alcohol with their children from a young age and felt well-prepared to have conversations about drinking because of information provided by school interventions: *“I don’t have a lot of questions on this topic, so I wouldn’t be so quick to visit it. I also had good information nights at my children’s schools. … So I’m pretty well equipped on that part”* (Parent 15, Netherlands).

#### Perceived Benefits and Barriers to Using FITSTART+

Parents mostly felt positive about FITSTART+’s resources, both in content and presentation. They felt the information was believable and reassured them of things they already knew. Further, parents mentioned that the videos were optimal lengths to maintain attention: *“I thought it was good that it was short and to the point. Because if the videos are too long, people will drop out, I think. I certainly would”* (Parent 12, Netherlands). Parents also received the PNF quiz favorably, with some commenting that receiving this feedback increased their motivation to explore FITSTART+ further: “*I started off with the survey first, and I thought it was an eye-opener for me. So then after that, I kind of branched off to read other things and find out more because from my results I realized I was a little off*” (Parent 7, Canada).

Parents identified a number of barriers that could prevent participants from maximally engaging with FITSTART+. First, some said the objectives of the web-app were not clear until after they complete the quiz. *“There’s quite a difference between the clarity of the information and the ease of use. It is easy to use, but not really clear about what it really wants or what the overall objective is”* (Parent 6, Germany). The amount of content and length of articles was also a barrier to some parents’ use of FITSTART+, but there was no clear consensus on how it could be improved. Some parents said the resource library had too much information (e.g., *“Everyone’s talking about how they would rather listen to these videos because the reading is too much”* [Parent 1, Canada]) and some found the articles to be too long, while others did not raise such concerns. A common criticism of the alcohol-related content, however, was that it focused too strongly on risks of alcohol use and did not provide tips on navigating difficult conversations within the same article: *“If you make a page that only focuses on harms you scare people off. There must be something from a more positive perspective. How do you touch these issues, how do you bring them to the table, how do you teach your child to have appropriate alcohol consumption?”* (Parent 6, Chile). Further, the scientific language was also discouraging for some parents: *“I think [the articles are] so scientific. Such as the ‘protective behavioral strategies’; you can also phrase that differently. This just doesn’t appeal.”* (Parent 5, Netherlands). For German participants, anonymity and a more caring approach were needed to make the web-app suitable to them. *“I haven’t got the patience and I’d be off within seconds. It would need to be much more anonymous, and a bit more of a caring approach to potentially understand the sensitive nature of why your child is having alcohol issues. I found it quite impersonal considering it is probably quite a complicated issue that you’re seeking help for”* (Parent 1, Germany). Parents from Canada, the Netherlands, and Germany raised concerns about the use of personal data: *“I only created a login and nothing else. And I would never go further than that with these kinds of websites. I don’t know who’s on it and I don’t like to share more information than necessary. I wouldn’t put a picture on it, or any information about my child.”* (Parent 14, Netherlands). They said that the mandatory profile creation and sharing of their information with other parents would discourage parents from using the platform: *“As soon as you start having to put in personal data and you don’t know why you’re putting in personal data, I’m turned off again”* (Parent 4, Germany); *“The tool is to help parents, so they shouldn’t have to log in to get help”* (Parent 13, Canada). Some even mentioned that data protection legislation in their countries would not allow some of the features in this intervention.

#### Adaptations to Increase Relevance to Parents’ Context

All parents recognized the importance of adapting the intervention to make it more relatable to the parents of their own countries, yet adaptation recommendations differed from parent to parent. The most repeated suggestion was mentioned in nine out of 11 focus groups, which was to add a section meant for adolescents themselves or a section for parents and adolescents to use together. “*It would be a nice feature to include a questionnaire that you could fill in together with your child, like what do you think and what do I think, and then you could get some sort of outcome to see how we both feel about it”* (Parent 2, Netherlands). In the same line of thought, parents mentioned that they would prefer to combine the information and facts given by experts with case studies, adolescents’ opinions, and role-playing scenarios between parents and children: *“if that had been more of a case study, say, of a parent and child talking about [the content], I would have liked it even more. But then I would say have both, something for everyone. So, the professor who says what research shows what the best conversation method is, and also a parent-child conversation or experience”* (Parent 7, Netherlands). Other suggestions were focused on ease of use and making the app more interactive. Parents suggested adding a search function to look for specific content in the web-app, linking other sites to find further information or professional help, including a podcast or adding a chat or forum section to connect with other parents, and sending notifications to parents when new content is released. Moreover, some parents suggested making the content more personalized, according to parents’ interests or their child’s age.

Another suggestion that appeared frequently was to change the way in which the alcohol content was presented. Parents from every country thought that the web-app had a strong perspective regarding parenting around alcohol that did not allow parents to make up their own minds: *“You said that the goal of the website is to help children avoid risk and I don’t know if that’s your goal as a parent. We might have a slightly different view of this in the Netherlands than in America. So, I would be very sorry if my children were to do very extreme things, but at the same time, it is of course the time in your life when you are going to discover things and make mistakes” (Parent 13, Netherlands).*

In general, Canadian parents had the most positive opinions of the web-app and had fewer comments about possible adaptations to their context. The main adaptation Canadian parents mentioned was changing the target group to parents of pre-adolescents and adolescents, as for them that is the most vulnerable time to prevent teenage drinking: *“I feel like parents need to know this information prior to the summer of grade nine because it’s a make-or-break summer for a lot of kids”* (Parent 1, Canada). This adaptation was also important to Chilean parents, who even added that there should be different content according to the child’s age: *“There should be age segmentation. For children who are younger there should be certain content, children of 13 who perhaps haven’t started drinking are very different from those of 19 or 17 who already drink” *(Parent 5, Chile). Chilean parents showed the greatest concern about teenage drinking problems in their country and thought the web-app could be beneficial only if it was part of a broader intervention about topics such as neglect, sexual development, self-care, drugs, peer pressure, and social media exposure: *“You could start with other topics like how to respect other people, respect nature, drinking and taking care of your body, [and] eating well. There are several topics and if you divide it up and say we have an application that will help you with the alcohol issue, but, well, alcohol is mixed with a lot of other things”* (Parent 1, Chile). Dutch parents also suggested adding information on a wider range of topics, especially consumption of other drugs such as cannabis and prescription drugs, which for them is a bigger issue than alcohol: *“it seems as if alcohol is still really taboo in America, while I am more concerned about drugs, for example those pills, or indeed sexuality. I would actually rather know more about that.”* (Parent 9, Netherlands). Dutch parents frequently mentioned that the cultural approach to alcohol is very different in the Netherlands compared to the U.S. They advocated for giving their children more independence to make their own decisions about alcohol than was present in FITSTART+: *“So I think indeed that the Dutch situation requires information, but a little more from a distance. Informing and facilitating parents, but not directing. I think the American aspect is very focused on directing the child’s life, and we’re not used to that here… in the Netherlands, we place that control in the hands of the children themselves. They also need to learn to stand on their own two feet”* (Parent 13, Netherlands). Therefore, the intervention’s zero-tolerance message was not culturally appropriate and Dutch parents found it moralistic and patronizing.

## Discussion

The current study had two aims: (1) examine the implementation of FITSTART+ with the target population by assessing parents’ responsiveness to intervention content and perceived intervention quality; and (2) identify ways in which this program could be adapted to successfully disseminate it to other parent groups who might benefit from a similar program (e.g., non-U.S. parents of adolescents). Overall, the target population was responsive to the program (i.e., the majority completed the PNF quiz, read the alcohol- and parenting-related resources, and indicated that FITSTART+ was easy to use and free of major technical issues). The quality of FITSTART+’s content was also rated positively, with nearly 90% of parents reporting they would recommend the intervention to other parents of incoming college students. Parents also found the website to be aesthetically pleasing and the parenting resources to be presented well. These parents’ overall positive reception is not surprising given that person-centered methods, which are shown to improve participants’ reception of intervention content (LaMonica et al., 2022; Noorbergen et al., 2021; Perski & Short, 2021; Yardley et al., 2015), were used during the creation of FITSTART+ by directly involving the target population in its development (e.g., branding, resource library content, alcohol-related norms included in PNF). U.S. parents also suggested several modifications to the web-app that might further improve parent responsiveness and perceived intervention quality in the future: adding a moderated forum, gradually releasing new content once per week, and providing parents more guidance within the program that could help them make the most of its resources.

The non-U.S. sample provided several cultural adaptations that they considered necessary to be made before the intervention would be relevant to them and their contexts. The most endorsed barrier to using the app, mentioned by parents from three out of the four countries, was being required to create a profile and provide personal information before accessing the program’s resources; many parents discussed anonymity as a necessary precursor to using the web-app. Stringent laws in the European Union which govern websites’ collection of personal information regulate what data can be collected by web-apps and what can be done with it; thus, foregoing the collection of any personal information may help to ensure European parents feel comfortable using the PBI. The most frequent suggestions for adaptations to FITSTART+, endorsed by parents from all countries, was to present the resource information in a neutral way without encouraging a zero-tolerance approach to alcohol use. This is not surprising given differences in the role of alcohol in daily life in the U.S. compared to other countries, particularly European ones (i.e., wet vs. dry cultures; Bloomfield et al., 2003). Other possible adaptations differed more significantly by country. For instance, Chilean parents suggested the intervention content should be embedded within a larger intervention focused on health behaviors more broadly. Dutch parents also mentioned providing a broader range of topics would be welcome. They also expressed the strongest concerns with the framing of the alcohol content, noting cultural and social differences around alcohol between the Netherlands and U.S. that must be considered before parents would use the web-app (e.g., Bloomfield et al., 2003). In sum, non-U.S. parents agreed cultural adaptation that considers factors such alcohol use norms, laws, and general attitudes toward alcohol consumption is essential for successful implementation.

### Implications

As previous studies have emphasized, our findings underscore that it is imperative to critically assess transferability of an intervention to different contexts prior to its implementation and dissemination (Movsisyan et al., 2019; Schloemer & Schröder-Bäck, 2018; Koning et al., 2021). When modifying an intervention to improve cultural fit and relevance, it is necessary to first have an intimate understanding of the target population’s social and cultural context so the content can be presented in a maximally appealing way. As such, a second-stage person-centered approach wherein insights are gathered from members of each new, country-specific target may be critical to ensure successful adaptation and eventual implementation. Parents’ preferences for the content of web-based interventions may differ largely across (cultural) contexts; therefore, researchers need to be proactive in adapting an intervention accordingly prior to its implementation.

Our findings also reveal insight into parents’ interactions with web-based PBIs. As our U.S. sample’s data suggest, it is not feasible that parents will engage with all aspects of an online intervention but will instead explore a subset of resources that most appeals to them. Therefore, PBI resources should be presented in a way that allows parents to engage with as much content as possible and does not create barriers to exploration. For instance, removing the account creation step of FITSTART+ may encourage more parents to view resources and caters to parents who may be minimally engaged with the program. For parents who are more motivated to utilize the program and are interested in advanced features (e.g., a forum to connect with other parents), creating an account to access these features would be unlikely to be seen as burdensome and would not hinder parents’ use. Therefore, providing a range of features is optimal to cater to all individuals and ensure parents engage with the intervention to the full extent they are willing to.

### Limitations and Future Directions

The current study had several limitations that should be addressed in future research. First, although we conducted more focus groups than what is shown to be necessary to identify 80–90% of emerging themes (Guest et al., 2017), future studies should use larger sample sizes and more diverse sampling methods to ensure parents’ suggestions for cultural adaptations to interventions are representative of their populations. A second limitation was that the non-U.S. sample only had 15 min to spend with the web-app before providing their feedback. This limited amount of time meant that parents likely did not explore all aspects of the application and were unable to read the included resources in depth. Future focus groups should permit parents more time explore the program before asking for feedback. Third, we did not obtain information on what kind of devices parents used to access the app (i.e., smartphone, tablet, or laptop/desktop computer). Although the app was adaptive to the device used and displayed content in a way that fit each individual screen, there are undoubtedly differences in app layout and user experience across devices (e.g., parents may not have wanted to spend as much time accessing FITSTART+ via a smartphone because it would require more scrolling to read an article). Future studies should explore what devices parents use to access web-based interventions, and if this affects engagement or acceptability of the PBI. Fourth, our non-U.S. sample and the countries those parents represented was a convenience sample. Nevertheless, the four countries represent populations with above-average rates of high-risk drinking among adolescents (WHO, 2018). Thus, the sampled countries are ideal targets for parent-based alcohol interventions as binge drinking is a concern among adolescents, the target population of the intervention. Finally, the age of the adolescents in the U.S. sample was somewhat older than in the non-U.S. sample, which although intentional, is likely to have affected non-U.S. parents’ perceptions of the relevance of FITSTART+’s content. Future studies are needed to examine age-matched groups to rule out if some of the cultural differences were due to differences in adolescents’ ages.

## Conclusion

This is the first study to report findings from a comprehensive process evaluation of a web-based PBI. Utilizing a mixed-method approach and both U.S. and non-U.S. samples, we found that parents’ responsiveness to FITSTART+ and the quality of the intervention’s content was rated positively among the population it was intended for (i.e., U.S. parents of incoming college students). Non-U.S. parents of adolescents provided a range of cultural factors that should be considered and that would be necessary to incorporate into an adapted version of FISTART+ for it to be well-received among these different populations. In conclusion, findings from this study illustrate that process evaluations are a vital step in informing the development and refinement of PBIs and involving parents in the intervention design process is critical, particularly when transferring an intervention outside of the context that it was original designed for.

## Footnotes

1 According to the WHO (2018), over half of current 15–19-year-old drinkers in each sampled country engaged in heavy episodic drinking (HED): 54.4% in Chile, 51.7% in Canada, 70.2% in Germany, and 60.9% in the Netherlands. This places Chile and Canada in the top 12% and 15%, respectively, of countries in the Americas with the highest rates of HED among this population. Similarly, Germany and the Netherlands are in the top 10% and 34%, respectively, among European countries (WHO, 2018).
